# The elite university paradox: physical literacy and its associated factors among 46,054 Chinese college students

**DOI:** 10.3389/fpubh.2026.1889306

**Published:** 2026-07-03

**Authors:** Rouna A, Xiaowei Zhu, Song Wang

**Affiliations:** 1 School of Marxism, University of Science and Technology Beijing, Beijing, China; 2 College of Marine Culture and Law, Jimei University, Xiamen, China; 3 Department of Physical Education, Peking University, Beijing, China

**Keywords:** college students, influencing factors, physical activity participation, physical literacy, policy implications

## Abstract

**Objective:**

Physical literacy is an important factor for individuals to maintain health. This study aims to investigate the status of physical literacy among Chinese undergraduate college students and its influencing factors.

**Methods:**

Utilizing national survey data on undergraduate college student participation in physical activity conducted by the Chinese Ministry of Education in 2024, a total of 46,054 participants were included. Linear regression analysis was employed to analyze the data.

**Results:**

The physical literacy of Chinese undergraduate college students is moderate. Female students, first-year students, and students from top-tier universities demonstrated relatively lower levels of physical literacy. Factors such as the exercise atmosphere on campus, the degree of attention to physical exercise, the accessibility of exercise facilities and guidance, sufficient exercise time, and the number of exercise partners were found to be associated with physical literacy of Chinese college students.

**Conclusion:**

Sustained attention should be devoted to enhancing physical literacy among Chinese university students, particularly those enrolled in top-tier universities. Efforts should be made to provide a good exercise environment, facilities, and guidance for college students and encourage them to exercise in groups and maintain a certain amount of exercise time.

## 1 Introduction

Physical literacy is a personal disposition that emphasizes the importance of lifelong physical activity (1). It can be described as the motivation, confidence, physical competence, and understanding of the value of physical activity that enables an individual to engage in lifelong physical activity (2). It is also a key component of physical education and individual physical and mental well-being (3). In recent years, physical literacy has garnered broad attention across various research fields (4). Educational organizations and researchers worldwide recognize that physical literacy should hold equal educational value to literacy and numeracy (5–7). Enhancing physical literacy is viewed as a critical opportunity to yield significant health benefits for both children and adults (8–10). Moreover, improving physical literacy may reduce the financial burden on healthcare systems (11, 12). Consequently, physical literacy has become an important focus in global physical education, health promotion, and social development. Improving physical literacy across populations holds significant social value and importance.

With increasing attention given to physical literacy, research in this area has become quite extensive. Numerous studies now indicate that physical literacy forms the foundation for lifelong engagement in physical activity (13–16). Physical literacy has emerged as both a focus and a prerequisite for physical activity, demonstrating a positive correlation with activity levels (17). Given that university students represent a crucial force in societal development and function, they are at a significant life stage, balancing family expectations; personal achievement goals; and the demands of study, work, and life (18). Consequently, the physical and mental health of university students has garnered significant attention, and much research has focused on their physical literacy. Studies have shown that physical literacy helps mitigate declines in quality of life among university students (19), enhances their subjective well-being and mental health (20, 21), and fosters lifelong habits of physical activity, further promoting their overall health (22).

However, although prior research has extensively documented the health benefits of physical literacy among university students, comparatively limited attention has been devoted to examining its current status and underlying determinants within this population. This gap is particularly salient in the context of East Asian societies, especially China, where the development of physical literacy unfolds within a sociocultural environment characterized by intense academic competition. Deeply rooted cultural norms often position academic achievement as the principal pathway to social mobility and familial honor, thereby prioritizing intellectual attainment over physical development. Within this framework, time and energy are frequently perceived as zero-sum resources. As students progress through the educational hierarchy, the escalating pressure to sustain academic excellence, particularly within elite institutions, may constrain opportunities for physical activity and engagement in health-promoting behaviors. Despite abundant sports resources and supportive campus sporting cultures, physical education in Chinese world-class universities is largely confined to the early undergraduate years. As academic, research, and career demands increase, the lack of sustained physical education support contributes to lower levels of sport participation among senior students, thereby increasing their risk of physical literacy decline (23). Crucially, this tension is not unique to the Chinese context. Academic workload is frequently cited as a primary barrier to physical activity and exercise worldwide (24). The stress stemming from dense coursework and high-performance expectations consistently suppresses health-promoting behaviors across diverse educational systems (25). Such structural tensions may inadvertently impede the development of physical literacy. Moreover, students’ healthy lifestyle behaviors and physical activity are shaped not only by educational demands but also by sociocultural expectations and broader environmental contexts (26). Consequently, physical literacy emerges not merely as an individual attribute, but as a construct jointly shaped by structural factors, encompassing both educational regimes and environmental settings.

This research gap is especially noteworthy given that China enrolls more than 40 million university students annually, yet empirical evidence regarding their levels of physical literacy and its associated factors remains limited. To address this deficiency, the present study draws upon large-scale survey data to systematically examine the characteristics and correlates of physical literacy among Chinese university students. By doing so, it aims to provide evidence-based recommendations to inform future interventions and policies designed to enhance physical literacy and, ultimately, promote both physical and mental well-being within this population.

## 2 Methods

### 2.1 Data sources

The data were derived from a nationwide survey on undergraduate students’ participation in sports, jointly organized by the Chinese Ministry of Education and Tsinghua University between March and April 2024. The survey was conducted in 170 universities across China, including institutions located in Beijing, Shanghai, Jiangsu, Zhejiang, Henan, and Inner Mongolia, with primary coverage in the eastern, central, and northern regions of the country. A total of 57,359 questionnaires were collected, of which 46,054 were deemed valid, yielding a valid response rate of 80.29%.

### 2.2 Measures

The physical literacy scale used in this study assesses participants’ perceived physical literacy across five key dimensions: motivational persistence, health-promotion intention, self-assessed physical fitness, sport skill competence, and scientific exercise knowledge. It is important to note that this instrument measures perceived components of physical literacy rather than providing a comprehensive and objective assessment of all dimensions of the construct. Physical literacy was assessed using five items: “I have the willpower to persist in exercise,” “I have the intention to improve my health through exercise,” “I possess good physical fitness,” “I can master at least one exercise skill,” and “I know how to exercise scientifically and efficiently.” All items were rated on a 5-point Likert scale ranging from 1 to 5, with higher scores indicating greater levels of agreement or perceived ability. The scale demonstrated excellent internal consistency, with a Cronbach’s alpha coefficient of 0.892. Confirmatory factor analysis further indicated a good model fit (CFI = 0.982, TLI = 0.965, SRMR = 0.025). These findings suggest that the scale possesses satisfactory reliability and construct validity.

The measurement methods for the other variables are as follows: Gender (1 = Male, 2 = Female), Grade (1 = First Year, 2 = Second Year, 3 = Third Year, 4 = Fourth Year), Major (1 = Natural Sciences, 2 = Arts and Social Sciences), Region (1 = Central, 2 = Northern, 3 = Eastern), Strong Exercise Atmosphere at the University (1 = Low--5 = High), University’s Emphasis on Exercise (1 = Low--5 = High), Easy Access to Exercise Facilities (1 = Low--5 = High), Having Many Exercise Partners (1 = Seldom to 5 = Many), Having Much Free Time for Exercise (1 = Seldom to 5 = High), Easy Access to Exercise Instruction (1 = Low--5 = High), and Availability of Digital Exercise Facilities at the University (1 = yes, 2 = No), University Type (1 = First Tier, 2 = Second Tier, 3 = Third Tier, 4 = Fourth Tier). It should be noted that regarding the classification of universities, first- tier universities refer to institutions designated under the national Project 985 and Project 211 frameworks, representing China’s most research-intensive elite institutions. Second-tier universities are provincially designated key undergraduate institutions. Third-tier universities are regular undergraduate degree-granting institutions. Fourth-tier institutions include independent colleges and those with a primarily applied or vocational undergraduate orientation. This tiered system reflects significant differences in institutional resources, research orientation, academic selectivity, and public prestige.

### 2.3 Statistical analysis

Statistical analyses were conducted via Stata 18.0. T tests and ANOVA were employed to assess significant differences across different levels. Additionally, we utilized linear regression models to examine the influencing factors of physical literacy.

## 3 Results

### 3.1 The state of physical literacy

Table 1 displays the characteristics of Chinese college students’ physical literacy. The results indicate that males scored higher on physical literacy than females did (*p* < 0.001). Undergraduate students in their fourth year demonstrated the highest levels of physical literacy (*p* < 0.001). Students majoring in the natural sciences had higher physical literacy scores than those majoring in arts and social sciences did (*p* < 0.001). Undergraduate students in the central region of China presented the highest levels of physical literacy (*p* < 0.001). Interestingly, students from universities rated as the lowest tier in China presented the highest levels of physical literacy (*p* < 0.001).

**Table 1:** Respondent characteristics.

Variable	Physical literacy		t/F	*p*
	*n* (%)	M ± SD		
Gender			36.52	< 0.001
Male	18,049 (39.19)	19.58 *±* 3.88		
Female	28,005 (60.81)	18.30 ± 3.54		
Grade			7.97	< 0.001
First year	23,459	18.84 ± 3.74		
Second year	16,187	18.70 ± 3.71		
Third year	4,851	18.90 ± 3.75		
Fourth year	1,557	19.02 ± 3.66		
Major type			9.18	< 0.001
Natural science	22,727	18.96 ± 3.80		
Art & social science	23,327	18.64 ± 3.65		
Region			24.58	< 0.001
Central	15,467	18.86 ± 3.68		
North	5,671	19.06 ± 3.71		
East	24,916	18.70 ± 3.76		
University type			65.71	< 0.001
First class	6,238	18.44 ± 3.70		
Second class	12,210	18.64 ± 3.68		
Third class	14,863	18.78 ± 3.77		
Fourth class	12,743	19.16 ± 3.71		
Total				
	46,054	18.80 ± 3.73		

### 3.2 Factors associated with physical literacy

Table 2 presents the results of the analysis of the factors influencing physical literacy among Chinese college students. Multicollinearity among predictors was assessed using Variance Inflation Factors (VIF). All VIF values were below 5.0, indicating that multicollinearity was not a concern in our models. The findings reveal that all factors except major and the availability of digital exercise facilities are significant predictors of physical literacy among college students. Specifically, males had higher physical literacy scores than females did (*p* < 0.001). Compared with those from the central region, students from universities located in the eastern and northern regions of China presented higher levels of physical literacy (*p* < 0.001). The oldest students demonstrated greater physical literacy than younger students did (*p* < 0.001). Furthermore, the results indicate that a stronger exercise atmosphere was significantly and positively associated with higher levels of physical literacy (*p* < 0.001). College students who had easier access to exercise facilities and guidance, more exercise partners, and sufficient time also presented higher levels of physical literacy (*p* < 0.001).

**Table 2:** Analysis of the influencing factors of physical literacy.

Variables	β	SE	t	*p*
Gender (1 = male)	−0.485	0.025	−19.50	< 0.001
Major type (1 = natural science)	0.028	0.024	1.12	0.261
Region (1 = central)				
North	0.188	0.031	6.14	< 0.001
East	0.296	0.041	7.18	< 0.001
University type (1 = fourth class)				
First class	−0.449	0.044	−10.27	< 0.001
Second class	−0.352	0.033	−10.74	< 0.001
Third class	−0.293	0.036	−8.14	< 0.001
Grade (1 = fourth year)				
First year	−0.225	0.063	−3.57	< 0.001
Second year	−0.169	0.064	−2.65	0.008
Third year	−0.084	0.069	−1.21	0.225
Strong exercise atmosphere [1–5]	0.472	0.023	20.70	< 0.001
Strong exercise attention [1–5]	0.401	0.016	24.81	< 0.001
Easy access to exercise facilities [1–5]	0.747	0.019	39.03	< 0.001
Many exercise partners [1–5]	0.770	0.022	35.16	< 0.001
Enough time for exercise [1–5]	0.489	0.016	29.94	< 0.001
Easy access to exercise guidance [1–5]	0.848	0.021	40.38	< 0.001
Have digital exercise facility (1 = yes)	−0.035	0.024	−1.44	0.149
Adj R^2^	0.594			

### 3.3 Regional heterogeneity analysis

Due to China’s vast geographical expanse and regional disparities in economic development, it is necessary to conduct a heterogeneity analysis of the factors influencing college students’ physical literacy from a regional perspective. Table 3 presents the results of this analysis. The findings indicate that, in the North and Central regions, university tier has a statistically significant positive effect on students’ physical literacy. However, in the East region, this effect is weakened and lacks statistical significance. Additionally, the impacts of other factors on college students’ physical literacy are largely consistent across regions, with no notable regional variations observed.

**Table 3:** Analysis of heterogeneity across different regions in China.

Variables	Central area			North area			East area		
	β	SE	*p*	β	SE	*p*	β	SE	*p*
Gender (1 = male)	−0.450	0.035	<0.001	−0.579	0.070	<0.001	−0.504	0.041	<0.001
Major type (1 = natural science)	0.021	0.036	0.552	0.200	0.071	0.005	−0.015	0.038	0.703
University type (1 = fourth class)									
First class	−0.426	0.062	<0.001	−0.895	0.153	<0.001	−0.270	0.142	0.058
Second class	−0.299	0.059	<0.001	−0.687	0.145	<0.001	−0.384	0.042	<0.001
Third class	−0.292	0.053	<0.001	−0.646	0.143	<0.001	−0.040	0.079	0.611
Grade (1 = fourth year)									
First year	−0.153	0.086	0.076	−0.445	0.166	0.007	−0.270	0.113	0.017
Second year	−0.116	0.087	0.185	−0.411	0.168	0.014	−0.154	0.114	0.178
Third year	−0.048	0.094	0.611	−0.143	0.183	0.436	−0.152	0.125	0.226
Strong exercise atmosphere [1–5]	0.430	0.032	<0.001	0.388	0.061	<0.001	0.621	0.039	<0.001
Strong exercise attention [1–5]	0.409	0.023	<0.001	0.490	0.046	<0.001	0.326	0.026	<0.001
Easy access to exercise facilities [1–5]	0.778	0.026	<0.001	0.590	0.053	<0.001	0.736	0.033	<0.001
Many exercise partners [1–5]	0.786	0.030	<0.001	0.630	0.060	<0.001	0.802	0.037	<0.001
Enough time for exercise [1–5]	0.479	0.022	<0.001	0.484	0.044	<0.001	0.505	0.029	<0.001
Easy access to exercise guidance [1–5]	0.789	0.029	<0.001	0.959	0.057	<0.001	0.895	0.035	<0.001
Have digital exercise facility (1 = yes)	−0.051	0.036	0.156	−0.004	0.069	0.955	−0.012	0.037	0.732
Adj R^2^	0.569			0.565			0.649		

## 4 Discussion

Our findings indicate that the overall physical literacy level of Chinese university students is moderate. Notably, students enrolled in lower-tier universities demonstrate higher physical literacy scores than their counterparts in top-tier institutions. This pattern can be understood within the broader institutional and socio-cultural context of China. At the institutional level, the hierarchical structure of the higher education system plays a critical role in shaping student priorities (27). Elite universities are typically characterized by an environment of intense academic competition, in which scientific research output, theoretical scholarship, and academic performance function as the dominant indicators of success. Within such a performance-oriented climate, students frequently perceive time and energy as limited resources. As a result, intellectual achievement is often prioritized, either consciously or unconsciously, over physical development, potentially constraining engagement in physical activity and the cultivation of physical literacy. Differences in educational orientation across university tiers may further reinforce this disparity. Lower-tier institutions often place relatively greater emphasis on applied skills, practical competence, and holistic student development. In contrast, top-tier universities tend to focus more heavily on rigorous scientific training and theoretical academic excellence. Within this institutional framework, physical literacy may be implicitly positioned as peripheral rather than integral to educational success, thereby reducing institutional support and student investment in physical development. This pattern is not unique to China. Evidence from elite U. S. universities suggests that physical education has increasingly occupied a marginal position within undergraduate curricula, highlighting a broader tension between academic excellence and physical development in highly competitive higher education environments (28). Beyond institutional factors, this phenomenon is also embedded in a broader cultural tradition. Educational traditions in New Zealand emphasize holistic well-being through the Hauora framework, in which physical, social, mental, and spiritual dimensions of health are viewed as interconnected (29). By contrast, in many highly competitive higher education systems, including elite universities in the United States and India, academic achievement often assumes a dominant position in students’ educational experiences. Historically, Chinese society has been influenced by a Confucian value hierarchy that privileges intellectual cultivation over physical prowess, often encapsulated in the notion of valuing the civil over the martial. This enduring cultural orientation has contributed to a social climate in which physical activity may be regarded as secondary to, or even in tension with, scholarly achievement. For students who have successfully navigated the highly competitive Gaokao to gain admission to elite universities, such values may be particularly internalized. While these individuals accumulate substantial academic cultural capital, they may simultaneously underinvest in physical capital and the development of lifelong physical activity habits. Taken together, these institutional and cultural dynamics help explain why students at top-tier universities exhibit comparatively lower levels of physical literacy. The findings further suggest that elite institutions should reconsider how physical health and physical literacy can be more fully integrated into their broader educational missions, particularly in light of their importance for sustainable learning and long-term well-being.

Our study revealed that male students score higher than female students do, older students score higher than younger students do, students from eastern and northern regions score higher than those from central China do. First, the higher physical literacy scores among male students than among female students may be attributed to gender differences in physical fitness and traditional societal expectations of gender roles in Chinese culture (30, 31). For example, males are often encouraged to engage in more physical activities from an early age, whereas females are more likely to be encouraged to pursue artistic or other non-sport activities. Similar gender disparities have been reported across diverse higher education contexts, including the United Kingdom, India, and several European countries, where female university students consistently encounter greater barriers to physical activity participation, such as academic demands, time constraints, and limited social support (32). These findings suggest that gender differences in physical literacy may reflect broader structural inequalities in opportunities for physical activity rather than merely individual preferences, highlighting a shared public health challenge across higher education systems globally. Second, regional disparities in physical literacy scores can be linked to economic imbalances across China. The central and western regions are economically less developed than the eastern coastal areas are, resulting in an unequal distribution of educational resources (33). This disparity allows more economically developed cities to attract better sports education resources (34), thereby advancing sports education and improving physical literacy among the local population.

Our findings also highlight that a strong sports atmosphere, increased emphasis on exercise for university students, and better access to exercise facilities are key factors in improving physical literacy. From the perspective of motivational system theory, environmental changes are associated with in group needs, which in turn generate intrinsic motivation (35). When students are immersed in an environment with a strong sports culture, this atmosphere may contribute to their sports participation behaviors through a sense of collective identity (36), which often fosters improved physical literacy. Additionally, the level of attention and investment in physical education by university management serves as an important indicator of the institution’s commitment to enhancing students’ physical literacy. In this process, university management plays a crucial role by providing the necessary resources for students to engage in physical activities and by encouraging participation through emotional support. The quantity, quality, and accessibility of exercise facilities on university campuses also directly impact the likelihood of student participation in physical activities. Universities with a wide range of high-quality exercise facilities can meet the diverse needs of the student body, thereby significantly enhancing students’ participation in sports and improving their physical literacy (37). Nevertheless, the present study found no significant association between digital exercise facilities and students’ physical literacy. A plausible explanation is that the adoption of digital exercise technologies remains limited across Chinese universities. Indeed, more than half of the respondents (52.53%) indicated that digital exercise facilities had not been incorporated into their physical education experiences. Furthermore, the effectiveness of digital exercise facilities depends not only on their availability but also on their meaningful integration into teaching and learning processes. High implementation and maintenance costs, together with insufficient instructional support, may constrain their educational impact. Consequently, the potential of digital exercise technologies to promote physical literacy has yet to be fully realized. These findings suggest that universities should not only continue improving conventional exercise environments but also strengthen the pedagogical integration of digital exercise technologies to better support students’ long-term engagement in physical activity.

Our research further suggests that having more exercise partners, easier access to exercise guidance, and more available free time are all positive factors in improving students’ physical literacy. First, encouragement and participation from friends, as well as invitations and praise, are significantly positively correlated with students’ engagement in physical exercise (38). Having more exercise partners means that students can receive more emotional support and mutual supervision, improving the continuity and effectiveness of their exercise routines. Second, professional guidance from physical education teachers is crucial in cultivating physical literacy. Such guidance helps improve the scientific basis and quality of students’ exercise behaviors, making their participation more effective (39). Finally, the availability of more free time is essential for students to choose exercise programs, collaborate with coaches and partners, and actively engage in sports activities. Together, these factors form a student-centered approach to enhancing physical literacy, helping to internalize students’ motivation for physical exercise and ensuring the improvement of their physical literacy (40). In conclusion, university administrators should encourage students to exercise together, assist them in finding partners with similar interests and habits, and provide them with discretionary time for physical activities, as well as access to high-quality sports guidance.

## 5 Conclusion

Our study indicates that the physical literacy of Chinese undergraduate college students is moderate, with female students, first-year students, and students from top-tier universities exhibiting relatively lower levels of physical literacy. Factors such as the exercise atmosphere on campus, the level of attention given to physical exercise, the accessibility of exercise facilities and guidance, sufficient exercise time, and the number of exercise partners are associated with the physical literacy of Chinese college students. Therefore, at the school level, providing a good exercise environment, facilities, and guidance for college students is recommended. The elite institutions should reconsider how physical health and physical literacy can be more fully integrated into their broader educational missions, particularly in light of their importance for sustainable learning and long-term well-being. At the student level, students should be encouraged to exercise in groups and maintain a certain amount of exercise time.

## 6 Limitations

Our study has several limitations. First, although the physical literacy scale was developed based on established conceptualizations and theoretical foundations of physical literacy and demonstrated satisfactory psychometric properties, potential measurement bias cannot be entirely ruled out. Moreover, the instrument was a self-constructed five-item scale assessing perceived physical literacy. While it exhibited strong internal consistency (Cronbach’s *α* = 0.892) and good model fit (CFI = 0.982, TLI = 0.965, SRMR = 0.025), it did not fully capture key dimensions of physical literacy, particularly objectively assessed physical competence and behavioral engagement. Consequently, the findings should be interpreted as reflecting perceived physical literacy rather than the broader multidimensional construct. Future research would benefit from employing more comprehensive and widely validated instruments, such as the Perceived Physical Literacy Instrument (PPLI).

Second, this study utilized data from a large-scale national survey organized by the Chinese Ministry of Education. Given the survey’s primary focus on educational settings and institutional characteristics, the available variables were largely concentrated on school-level factors. As a result, potentially important determinants of physical literacy at the individual, family, and environmental levels were not adequately captured. Factors such as genetic predispositions, psychological characteristics, family socioeconomic background, previous physical education experiences, personal health history, seasonal variation, and climatic conditions may contribute substantially to physical literacy development but were unavailable in the dataset. This limitation is also reflected in the adjusted R^2^ value of 0.594, indicating that approximately 40.6% of the variance in physical literacy remained unexplained. Future studies should incorporate these broader determinants to develop a more comprehensive and internationally generalizable framework for understanding physical literacy among university students.

Third, the cross-sectional design precludes any inference of causal relationships among the observed variables. Longitudinal or experimental studies are needed to establish temporal ordering and causal mechanisms. In addition, the sample was primarily drawn from central, eastern, and northern China, which may limit the generalizability of the findings to western and northeastern regions, where climatic, economic, cultural, and demographic conditions differ substantially.

Finally, although environmental factors such as exercise atmosphere and facility accessibility were measured at the individual level, the data possess an inherently nested structure, with students clustered within universities. The use of ordinary least squares regression rather than hierarchical linear modeling may have resulted in underestimated standard errors and inflated statistical significance. Future research should employ multilevel analytical approaches to account for institutional clustering and to better distinguish individual-level and contextual influences on physical literacy.

## Data Availability

The raw data supporting the conclusions of this article will be made available by the authors, without undue reservation.
